# Supplementary material for: Predictors of Hypoxemia and Related Adverse Outcomes in Patients Hospitalized with COVID-19: A Double-Center Retrospective Study
Source: J Clin Med. 2021 Aug 14;10(16):3581. doi: 10.3390/jcm10163581 (PMC8397029; doi:10.3390/jcm10163581)
Supplement: Supplementary file 1 [file jcm-10-03581-s001.zip › jcm-1284799-supplementary.pdf]

Supplementary Materials

Predictors of Hypoxemia and Related Adverse Outcomes in Patients Hospitalized With COVID-19: A Double-Center Retrospective Study

Rabea Asleh, Elad Asher, Oren Yagel, Tal Samuel, Gabby Elbaz-Greener, Arik Wolak, Ronen Durst[1], Eli Ben-Chetrit, Ran Nir-Paz, Yigal Helviz, Limor Rubin, Ariella Tvito, Michael Glikson, and Offer Amir.

Supplementary Results

Association between BSA versus BMI and oxygen saturation in hospitalized COVID-19 patients

Morbid obesity has been shown to be an important risk factor for respiratory infections and for severe COVID-19 [1,2]. Although body surface area (BSA) has become a robust measure for indexing hemodynamic parameters, its prognostic value as compared to body mass index (BMI) in patients hospitalized with COVID-19 has not yet been studied. Therefore, we further sought to examine our hypothesis that BSA is positively correlated with COVID-19 severity as detected by low oxygen saturation levels in patients hospitalized with COVID-19. Moreover, we examined the differential associations of BSA compared with BMI in predicting low SpO_2.

When analyzed as continuous variables, BSA strongly correlated with BMI as expected ($r=0.75$; $p<0.001$) (Figure S1). We found that both BSA and BMI significantly but weakly correlated with oxygen saturation levels ($r=0.20$, $p<0.0001$ and $r=0.26$; $p<0.0001$, respectively) (Figure S2). Patients were then stratified by the median values of BSA (low; BSA<1.9 and high; BSA≥1.9) and BMI (low; BMI<27 and high; BMI≥27). Patients with high BSA showed significantly lower oxygen saturation (SpO_2) as compared to those with low BSA values (92.1 ±

5.2 vs. 94.0 ± 3.8; p<0.001) (Figure S3A). Similarly, patients with higher BMI had significantly lower SpO_2 compared with patients with low BMI (92.0 ± 5.3 vs. 94.1 ± 3.6; p<0.001) (Figure S3B). Patients who presented with both high BSA and high BMI had the lowest oxygen saturation percentages (91.8% ± 3.5%, n=193) when compared to patients with high BSA and low BMI (93.4% ± 4.2%, n= 52) or high BMI with low BSA (92.8% ± 4.8%, n=53), while those presented with both low BSA and low BMI had the highest SpO_2 values (94.2% ± 3.5%, n=194) (p<0.001 by ANOVA). When comparing differences between these 4 subgroups, we found that patients admitted due to COVID-19 and had their BSA and BMI both elevated, had significantly lower oxygen saturation than those with both low BSA and BMI measurements (p<0.001 using Tukey`s post hoc analysis), while no significant differences in SpO_2 were observed comparing other subgroups (Figure IV in Supplementary Materials).

References

1. Cai, Q.; Chen, F.; Wang, T.; Luo, F.; Liu, X.; Wu, Q.; He, Q.; Wang, Z.; Liu, Y.; Liu, L.; et al. Obesity and COVID-19 Severity in a Designated Hospital in Shenzhen, China. *Diabetes care* **2020**, *43*, 1392–1398.

2. Yang, J.; Hu, J.; Zhu, C. Obesity aggravates COVID-19: A systematic review and meta-analysis. *Journal of Medical Virology* **2021**, *93*, 257–261.

Supplementary Figures

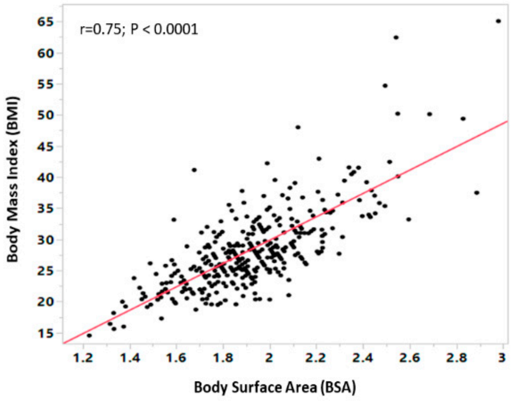

Figure S1. Correlation between BSA and BMI in hospitalized COVID-19 patients. BSA is presented in m^2, and BMI in kg/m^2. Pearson correlation coefficient; r=0.75, p<0.0001, N=492.

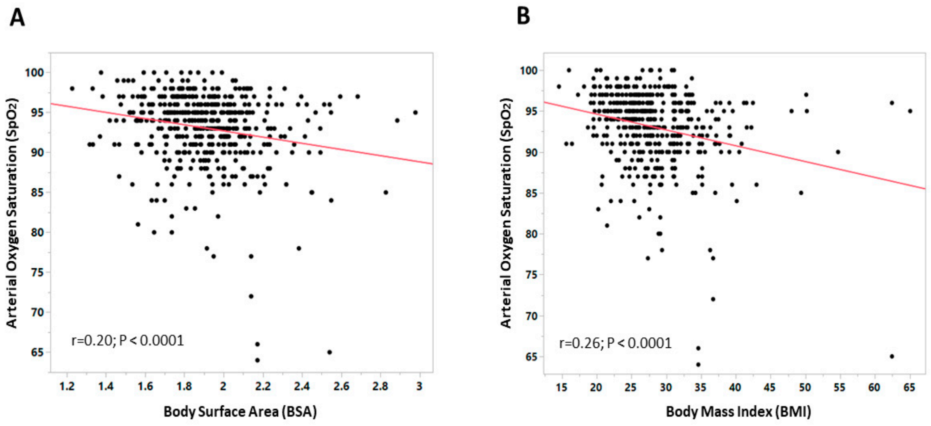

Figure S2. Correlations of BSA (**A**) and BMI (**B**) with oxygen saturation (SpO_2). SpO_2 is presented in percentages, BSA in m^2, and BMI in kg/m^2. Pearson correlation coefficient; r=0.20, p<0.0001 for BSA and r=0.26 for BMI, p<0.0001, N=492.

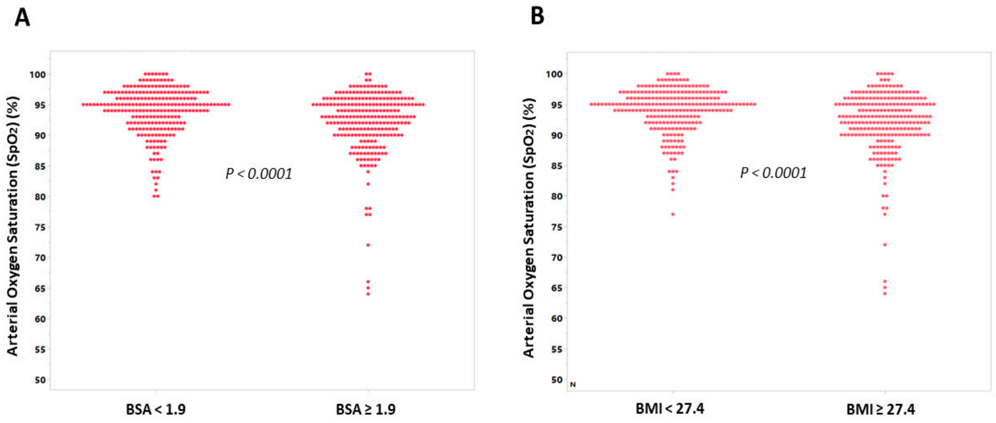

Figure S3. Association of body habitus with arterial oxygen saturation (SpO_2) in hospitalized COVID-19 patients. (**A**) SpO_2 was significantly lower in patients with high BSA than those with low BSA (92.1%±5.2% vs. 94.0%±3.8%; n=492; p<0.0001). (**B**) SpO_2 was significantly lower in patients with high BMI than those with low BMI (92.0%±5.3% vs. 94.1%±3.6%; n=492; p<0.0001).

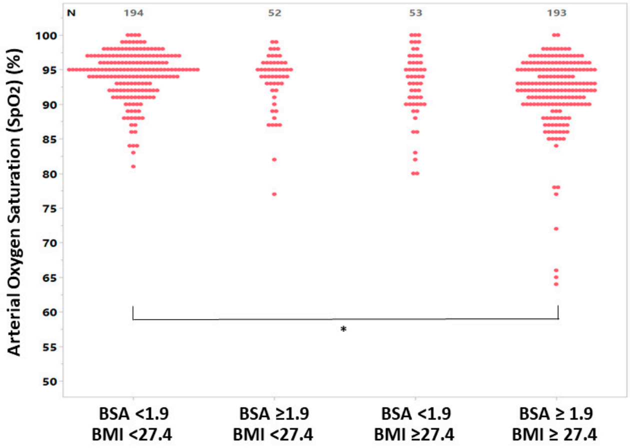

Figure S4. Differences in arterial oxygen saturation (SpO_2) among hospitalized COVID-19 patients stratified according to their BSA and BMI measures. SpO_2 is presented in percentages.

The mean±SD of SpO_2 was 94.2%±3.5% (n=194), 93.4%±4.2% (n=52), 92.8%±4.8% (n=53), and 91.8%±5.4% (n=193) for low BSA and BMI, high BSA and low BMI, low BSA and high BMI, and high BSA and BMI, respectively; $p<0.0001$ for the whole model by one-way ANOVA analysis. $P<0.001$ for subgroup analysis of SpO_2 in patients with high BSA and BMI vs. those with low BSA and BMI subgroups by Tukey`s post hoc analysis.

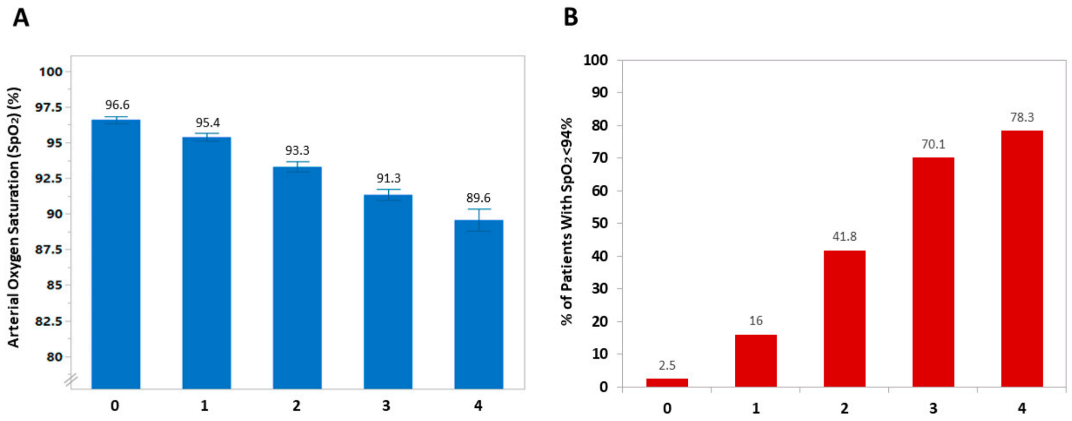

Figure S5. (**A**) Arterial oxygen saturation (SpO_2) according to the number of risk factors (age ≥50 years, BSA ≥1.9 m^2, lymphocyte count <1,400 cells/ul, and CRP ≥4 mg/dl) in each patient at the time of hospitalization with COVID-19. (**B**) The proportion of patients with SpO_2 <94% according to the number of these risk factors.
